# Evaluation of physicochemical properties, antioxidant potential and baking quality of grain and flour of primitive rye (*Secale cereale* var. *Multicaule*)

**DOI:** 10.1007/s13197-019-03827-1

**Published:** 2019-06-10

**Authors:** Małgorzata Warechowska, Józef Warechowski, Józef Tyburski, Ewa Siemianowska, Agnieszka Nawrocka, Antoni Miś, Marta Skrajda-Brdak

**Affiliations:** 1 Department of Systems Engineering, Faculty of Technical Sciences, University of Warmia and Mazury, Heweliusza 14, 10-718 Olsztyn, Poland; 2 Department of Process Engineering and Equipment, Faculty of Food Sciences, University of Warmia and Mazury, Oczapowskiego 7, 10-957 Olsztyn, Poland; 3 Department of Agroecosystems, Faculty of Environmental Management and Agriculture, University of Warmia and Mazury, Pl. Łódzki 3, 10-719 Olsztyn, Poland; 4 Institute of Agrophysics, Polish Academy of Sciences, Doświadczalna 4, 20-290 Lublin, Poland; 5 Chair of Food Plant Chemistry and Processing, Faculty of Food Sciences, University of Warmia and Mazury in Olsztyn, Pl. Cieszyński 1, 10-957 Olsztyn, Poland

**Keywords:** Primitive rye grain, Primitive rye flour, Physicochemical properties, Flour baking quality, Free phenolics, Antioxidant activity

## Abstract

The consumers interest in organic food and farmers’ search for cultivars with increased usefulness for organic farming have contributed to the revival of ancient cereal species and their launch onto the food market. In view of the above, the aim of this study was to determine the physicochemical properties, antioxidant potential and baking quality of grain and flour of primitive rye (*Secale cereale* var. *Multicaule* Polish: Krzyca), and to compare these parameters with open-pollinated and hybrid cultivars of common rye. The following determinations were made: the morphological and mechanical properties of grain, milling energy and the protein, starch, ash and free phenolic content of the analyzed flours, their amylograph characteristics and antioxidant potential. It was found that primitive rye has shorter kernels, lower thousand-kernel weight and a higher contribution of redness in color compared with common rye. In primitive rye grain rupture force was determined at 68.9 N and rupture energy at 35.6 mJ. Flours made from primitive rye grain have a higher content of ash and free phenolic compounds, lower starch content and similar antioxidant potential relative to common rye flours. The results of the amylograph test revealed that primitive rye flours were characterized by high baking quality. The primitive rye flours can be alternative ingredients for bread making and provide health advantage such as higher content of phenolic compounds. However, further research is needed to analyze variations in the properties of primitive rye grain and flour resulting from changes in environmental and climatic conditions.

## Introduction

Sustainable production and consumption plays a very important role in the food industry. The growing demand for food leads to agricultural intensification and increased use of chemicals (crop protection agents, fertilizers) which exert negative effects on the environment. The popularity of organic food is on the rise due to growing levels of consumer awareness about the environmental impacts of agricultural production. Consumers are also becoming increasingly health-conscious and seek organic foods that deliver health benefits (Dias et al. 2015).

Rye is a traditional crop in Central and Eastern Europe. Milled rye grain is used mainly in the production of bread as well as other processed foods, such as biscuits, breakfast cereals, muesli, pasta and extruded foods (Arendt and Zannini 2013).

Rye bread is a rich source of biologically active compounds, including antioxidants and dietary fiber, mainly arabinoxylans (Zieliński et al. 2008; Banu et al. 2010; Pihlava et al. 2018; Jonsson et al. 2018). It has been shown that the consumption of rye bread and other rye products (primarily as wholegrain products) provides health benefits (Jonsson et al. 2018; Koistinen et al. 2018). Rye from primary and secondary centers of origin in Transcaucasia and areas east of the Caspian Sea spread to northern Europe as a wheat weed. Primitive rye was more resistant to the cold winters and the nutrient-deficient and acidic soils of northern Europe than wheat, and with time, it became the main breadmaking cereal in this region. Primitive rye (*S. cereale var. Multicaule,* Polish*: Krzyca*) can be grown under difficult soil conditions. Its’ Polish (*Krzyca, Ikrzyca, Skrzyca*) and Czech and Slovak (*Kribica*) names denote a spark, and they were coined in an era of fire-fallow cultivation when arable fields were created by burning forests (Michalova 2000). Primitive rye was appreciated for its high yield and resistance to very difficult environmental conditions. In the past, flour made from primitive rye was not highly valued on account of its dark color which was associated with poverty rather than high nutritional value. However, primitive rye flour was recognized for its sweet taste and the prolonged freshness of bread (Podyma et al. 2013). Primitive rye is also known as midsummer rye because it was sown on Midsummer’s day. Early-sown rye was first cut as green fodder for cattle, and then left in the field over winter to produce grain in the spring. Primitive rye has never been modified through breeding, and it is an endangered species. The introduction of programs aiming to protect relict species (genetic biodiversity) has increased the availability of primitive rye seeds for organic farming. Primitive rye is generally grown on the poorest soils, and it is characterized by lower yields (1.5 to 2.5 t ha^−1^) than open-pollinated and hybrid rye cultivars for breadmaking.

According to the results of investigations, the demand for organic foods is on the rise (Dias et al. 2015). On the other hand, the food market is highly competitive, which forces producers to search for new food ingredients. Primitive rye could be an alternative ingredient in the milling and baking industries. Its grain is more abundant in free phenolics, flavonoids, sterols, tocopherols and carotenoids than common rye grain (Konopka et al. 2017b). This suggest higher health-promoting properties of Krzyca. The grain of primitive rye is characterized by a higher share of the seed coat, and the resulting bread has a sweet and honey-like aroma which is highly valued by consumers (Konopka et al. 2017b). In the baking of primitive rye breads is used sourdough fermentation by lactic acid bacteria. Sourdough fermentation causes reduction the phytates contents thanks to which it contributes to the increasing the bioavailability of minerals (Koistinen et al. 2018). In addition the rye breads on sourdough has been shown better antioxidative properties compared to wheat bread (Banu et al. 2010; Konopka et al. 2014). Flour from primitive rye grain poses an attractive alternative to common rye flour. However, the physical properties of grain (in particular properties that are essential for designing milling systems) and the baking quality of flour have to be determined before primitive rye can be used in the milling and baking industries. The relevant information is scant in the literature. However, Vejražka et al. (2011) investigated the physical properties and milling quality of *S. cereale* var. *Multicaule* cv. Lesan. The *S. multicaule* grain did not differ in physical properties from standard cultivars rye (*S. cereale*), but cv. Lesan, showed lower milling yields. The baking quality, nutritional value and health-promoting properties of primitive rye flour have been investigated by Konopka et al. (2017b). No published data is available on the physical, milling quality, flour quality and antioxidant potential of Primitive rye (*S. cereale* var. *Multicaule)* genotype Krzyca.

The aim of this study was to determine the physicochemical properties, antioxidant potential and baking quality of grain and flour of primitive rye (*S. cereale* var. *Multicaule*), and to compare these parameters with open-pollinated and hybrid cultivars of common rye (*S. cereale* L.). These studies can contribute to the popularization of primitive rye genotype Krzyca.

## Materials and methods

### Plant material

The experiment was performed on organically grown grain of primitive rye (*S. cereale* var. *Multicaule)*. The evaluated genotype was obtained by breeding grain from the collection of the Plant Breeding and Acclimatization Institute—National Research Institute in Radzików, Poland. Two organically grown cultivars of common rye (*S. cereale* L.), including open-pollinated rye cv. Dańkowskie Złote and hybrid rye cv. KWS Bono, were used as the reference standards. Open-pollinated rye cv. Dańkowskie Złote was placed on the Polish National List of Agricultural Plant Varieties (COBORU) in 1968 and remains the oldest registered rye variety, which best testifies to its unique properties. Hybrid rye cv. KWS Bono was registered in Poland in 2014. Primitive rye and both common rye cultivars were grown in an organic farm in Godki near Olsztyn in north-eastern Poland (53°49′53″N, 20°14′20″E).

### Grain analysis

The qualitative parameters of grain were evaluated with the use of standard methods. The moisture content of rye kernels was determined based on PN-EN ISO 712: 2012 method, kernel weight was determined based on AACC method 55-31 (2002). Thousand-kernel weight was determined for each sample with the use of an electronic kernel counter (Kernel Counter LN S 50A, UNITRA CEMI, Poland) and an electronic scale (WPE 120, Radgwag, Poland, d = 2 mg). The geometric parameters of grain were measured manually with an electronic caliper (Δ = ± 0.05 mm). Grain and flour color was analyzed with the Minolta CR 400 Chroma Meter (CR 400, Konica Minolta, Japan). Grain color was expressed by parameters L*, a* and b* of the CIELAB system. Individual kernels were subjected to a quasi-static compression test in the AXIS system (Poland) equipped with the FB-500 dynamometer with a measuring range of 0–500 N and a vertical stand with STAV power drive system. Compression force and head displacement were registered by a PC connected to the device. Every kernel was placed between two parallel plates and compressed with a constant velocity of 0.1 mm min^−1^ until the achievement of a fixed distance of 0.3 mm between the plates. The mechanical properties of grain were expressed by rupture force (F_r_) and force at the end of compression (F_e_). Rupture energy (E_rc_) and total compression energy (E_t_) were calculated (Dziki et al. 2014). The compression test was performed on 30 randomly selected rye kernels, and the arithmetic average of the measured parameters, were calculated for each cultivar.

### Grain milling and grain milling energy

Two types of flour were produced in the milling test: extracted flour and wholemeal flour. The moisture content of grain was brought up to approximately 15% (on a wet basis), and grain was ground in the Quadrumat Junior roller mill (Brabender^®^, Germany). The sifting roller was removed from the mill to obtain middlings (wholemeal). Kernel samples (125 g) from each rye genotype/cultivar were weighed to the nearest 10 mg on the WLC 2/A1 electronic scale (Radwag, Poland) and milled in the laboratory. Twenty-five samples of each genotype/cultivar were milled. The specific milling energy E_r_ (kJ/kg) was calculated with the following formula: E_r_ = (E_c_ − E_s_)/m_g_, where: E_c_—total energy consumed by the mill; E_s_—energy required for initiating the motion of ground particles (E_s_ was calculated by multiplying active power in idle mode by milling time); m_g_—mass of milled sample (kg). Fifteen replicates of middlings from each rye cultivar were separated with a sieve shaker (Analysette3^®^Fritsch, Germany) equipped with sieve 200 μm openings and a collection pan. The sample was shaken for 10 min (vibration amplitude of 1.5 mm). The fraction that passed through the 200 µm sieve was regarded as extracted flour. Flour yield was determined as the percentage of straight-grade flour.

### Particle size analysis

The size distribution of flour particles was determined by laser diffraction analysis (LDA) using the Malvern Mastersizer 2000 analyzer (version 5.22, Malvern Instruments, Malvern, UK) according to the AACC 55-40-01 (2002). The arithmetic average size of particles in extracted, and wholemeal flours were calculated. The average size of flour particles was determined by summing up the products of particle size (*d*_*i*_) and volume fraction (*φ*_*i*_): *d*_*avg*_ = SUM(*φ*_*i *_*·d*_*i*_). The size of flour particles d(0.1), d(0.5) and d(0.9) (µm) corresponding to the maximum diameter of 10%, 50% and 90% of flour particles, respectively, was determined. The span of volume-based size distribution was determined as follows: SPAN = (d(0.9) − d(0.1))/d(0.5).

### Flour and dough analysis

The flours were evaluated for ash content (ICC method 104/1, ICC 1990) and protein content (Kjeldahl method; KjelFlex K-360 distillation unit, Büchi, Germany; N·5.7). Starch content was determined by the polarimetric method according to Standard PN-EN ISO 10520: 2002 with the Carl Zeiss Jena 730083 polarimeter with the PGH Rundfunk-Fernsehen type G power transformer. The activity of α-amylase was determined in the Falling Number Apparatus 1800 (Petren, Sweden) in the Hagberg-Petren test according to Standard PN-EN ISO 3093: 2010. The amylograph test was conducted in a Brabender amylograph (type 800145) according to Standard PN-EN ISO 7973: 2016-01. The water absorption at dough consistency of 500 BU were determined according to a standard procedure (ICC 115/1, ICC 1992) with use Farinograph-E (Brabender, Duisburg, Germany).

### Preparation of rye flour extracts

The antioxidant activity and the free phenolic content of flours were determined in flour samples extracted thrice with 80% methanol. Flours were extracted in the MSC-100 thermo-shaker incubator (Hangzhou Allsheng Industries) for 15 min at 1400 rpm and a temperature of 22 °C. The supernatant was separated in an Eppendorf shaker (type 5810R) for 10 min at 10,000 rpm and a temperature of 22 °C.

### Determination of free phenolic content

The content of free phenolic compounds was determined spectrophotometrically with the Folin–Ciocalteau reagent (Merck) according to Konopka et al. (2012). The color reaction was carried out by adding the Folin–Ciocalteau reagent (0.5 mL), 14% sodium carbonate (3 mL) and distilled water (6.5 mL) to the polyphenol extract. After mixing, the solution was left for 60 min, and absorbance was measured against the reagent sample (without the phenolic extract) at a wavelength of 720 nm with the UNICAM UV/Vis UV2 (ATI Unicam, Cambridge, UK) spectrophotometer. The content of free phenolic compounds was expressed as mg D-catechin (Sigma Aldrich) equivalent per 100 g of sample dry mass.

### Antioxidant analysis

The antioxidant potential of flour samples was determined in the DPPH (2,2-diphenyl-1-picrylhydrazyl) radical scavenging assay according to Konopka et al. (2017a). The extracts were added to a DPPH (Sigma Aldrich) solution (0.2 mmol L^−1^ in methanol), and the mixture was shaken and incubated in the dark at room temperature for 30 min. Absorbance was measured at 517 nm against methanol, using FLUOstar Omega multi-mode microplate reader (BMG LABTECH, Offenburg, Germany). Antioxidant capacity was determined based on a curve of % DPPH·scavenging activity of different Trolox (Sigma Aldrich) concentrations in methanol and expressed as µmol Trolox per 100 g of flour dry mass.

### Statistical analysis

The results were processed statistically using Statistica for Windows v. 10 software (StatSoft Inc.). The data were analyzed by one-way analysis of variance (ANOVA). The significance of differences between means was determined by Tukey’s test (*p* ≤ 0.05). The data were also subjected to Pearson correlation coefficient analysis. The results were regarded as statistically significant at *p* ≤ 0.05.

## Results and discussion

### Physicochemical properties of grain

The physical properties of the grain of primitive rye and common rye cv. Dańkowskie Złote (open-pollinated cultivar) and cv. KWS Bono (hybrid cultivar) are presented in Table 1. The thousand-kernel weight (TKW) of primitive rye was low (21.6 g) compared with common rye cultivars, and its bulk density was determined at 756.3 kg m^−3^. The kernels weight is dependent on the growth conditions and cultivar (Järvan et al. 2018). Primitive rye grain did not differ significantly in width (2.48 mm) from the grain of open-pollinated and hybrid cultivars, but it was characterized by smaller average kernel length (6.69 mm). The average thickness of primitive rye kernels (2.00 mm) was similar to the thickness of hybrid rye kernels and significantly smaller than the thickness of open-pollinated rye kernels. The kernels of the evaluated rye cultivars were characterized by considerably smaller width and thickness than the grain analyzed by Jouki et al. (2012), where the above parameters were determined at 4.65 mm and 3.18 mm on average, respectively.

**Table 1:** Physical properties of primitive rye and common rye grain

Parameter	Genotype/cultivar		
	Primitive rye	Dańkowskie Złote (OP*)	KWS Bono (H)
Moisture content (% w.b.)	14.96^ab^ ± 0.03**	14.94^a^ ± 0.04	15.08^b^ ± 0.07
TKW (g)	21.6^a^ ± 0.9	28.5^b^ ± 1.5	28.1^b^ ± 0.8
TW (kg m^−3^)	756.3^b^ ± 1.7	749.1^a^ ± 1.6	771.0^c^ ± 4.5
Grain dimensions			
Length (mm)	6.69^a^ ± 0.80	7.74^b^ ± 0,77	7.40^b^ ± 0.91
Width (mm)	2.48^a^ ± 0.23	2.53^a^ ± 0.33	2.42^a^ ± 0.29
Thickness (mm)	2.00^a^ ± 0.21	2.21^b^ ± 0.17	1.98^a^ ± 0.31
Color parameters			
L*	51.3^b^ ± 2.2	54.3^c^ ± 2.8	47.8^a^ ± 2.4
a*	5.24^c^ ± 0.74	4.24^b^ ± 0.90	3.29^a^ ± 0.37
b*	19.04^ab^ ± 1.22	19.88^b^ ± 1.07	17.99^a^ ± 1.20
Rupture force, F_r_ (N)	68.9^a^ ± 28.9	83.7^ab^ ± 24.1	88.8^b^ ± 35.5
Force at the end of compression, F_e_ (N)	95.3^a^ ± 21.3	96.1^a^ ± 25.0	106.1^a^ ± 29.7
Rupture energy, E_rc_ (mJ)	35.6^a^ ± 22.4	37.7^a^ ± 23.3	42.5^a^ ± 24.7
Total compression energy, E_e_ (mJ)	81.5^a^ ± 26.5	79.9^a^ ± 22.5	90.6^a^ ± 33.7
Specific energy of milling E_r_ (kJ kg^−1^)	95.3^b^ ± 2.5	84.8^a^ ± 2.3	123.1^c^ ± 17.1

*OP open-pollinated cultivar, *H* hybrid cultivar

**Values given as the average values ± standard deviation (SD). Values marked with the same letters in rows for the same type of flour are not significantly different at *p* ≤ 0.05

In the color analysis, the average value L* of primitive rye grain (51.3) was significantly higher than in hybrid rye (47.3) and lower than in open-pollinated rye (54.3). The grain of primitive rye was characterized by a higher contribution of redness (a* = 5.24) in comparison with the grain of common rye cv. Dańkowskie Złote (a* = 4.24) and KWS Bono (a* = 3.29), but it did not differ significantly from the reference rye in the value of the b* (19.04). According to Zykin et al. (2018), the color of cereal grain is determined mainly by the content of anthocyanin pigments.

The mechanical properties of cereal grain play a very important role in the milling process (Ponce-García et al. 2016). The force needed to deform grain has to be accurately determined for the purpose of designing grain harvesting and processing equipment. In primitive rye grain, rupture force was determined at 68.9 N, force at the end of compression—at 95.3 N, rupture energy—at 35.6 mJ, and total compression energy—at 81.5 mJ (Table 1). The rupture force of primitive rye grain was significantly lower compared with the open-pollinated cultivar. The remaining parameters (F_e_, E_rc_ and E_e_) did not differ significantly across the analyzed cultivars. The average specific milling energy of primitive rye grain (95.3 kJ kg^−1^) was approximately 11% higher relative to the open-pollinated cultivar and approximately 29% lower relative to the hybrid cultivar. Milling energy is determined by the milling method and the properties of grain, mainly moisture content, kernel size and hardness and the degree of milling (Dziki et al. 2014; Warechowska et al. 2016). In a study by Rydzak et al. (2012), energy consumption during the milling process of a mixture of rye grain with similar moisture content, ground in the same type of a mill, was higher than that noted for primitive rye grain in our study. In the work of Hameed Hassoon and Dziki (2017), the milling energy of rye grain ground in a hammer mill ranged from 66.2 kJ kg^−1^ (grain with 10% moisture content) to 133.6 kJ kg^−1^ (grain with 18% moisture content).

### Particle size distribution of flour

**Fig. 1:**
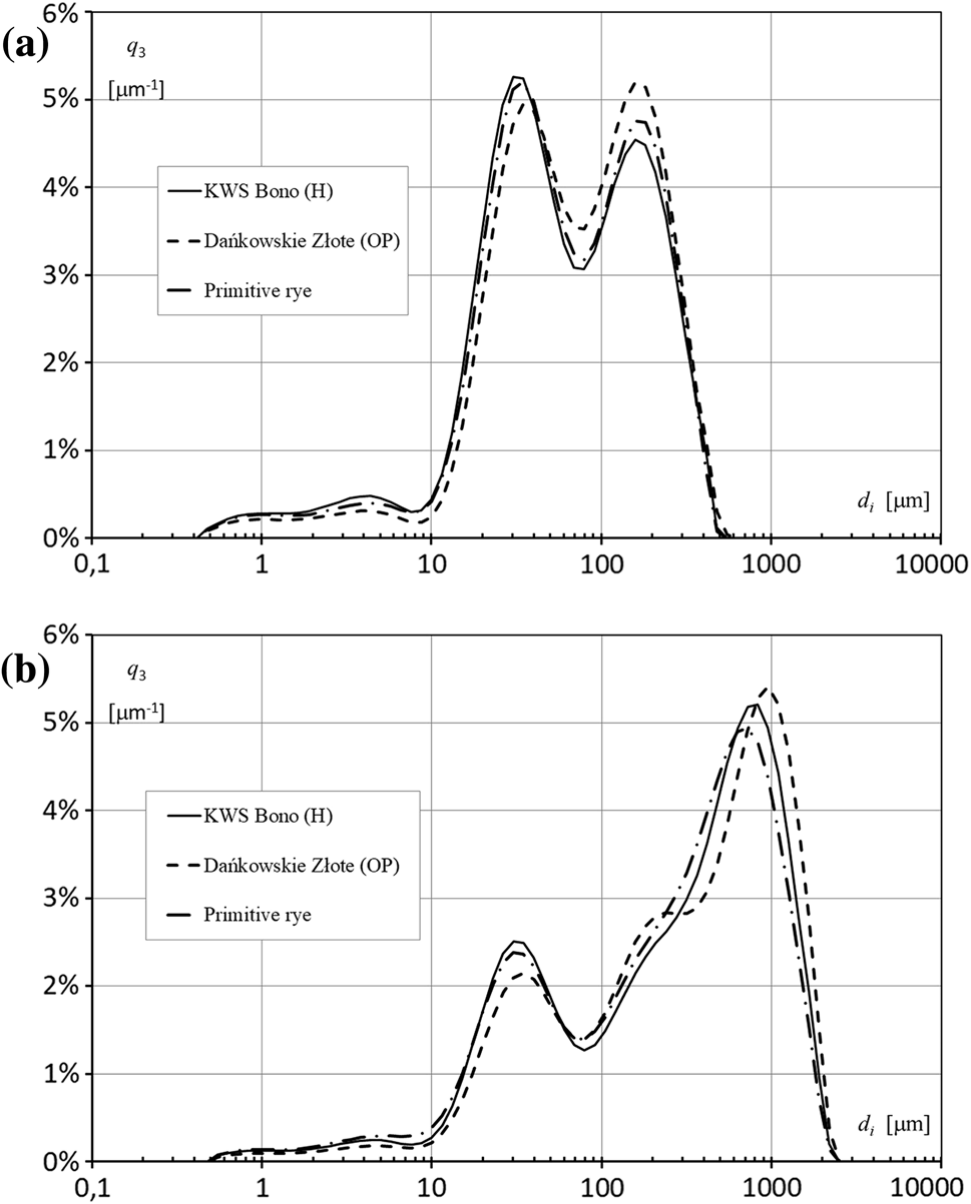
The granulometric composition of flours from the grain of: primitive rye, common rye cv. Dańkowskie Złote (OP) and common rye cv. KWS Bono (H); **a** extracted flour; **b** wholemeal flour

The curves presenting the particle size distribution of extracted and wholemeal flours are shown in Fig. 1. All flours had quatrimodal size distribution with a trace amount of fractions smaller than 10 μm. The average particle size of wholemeal flour (74.8 µm) and extracted flour (73.8 µm) from primitive rye grain was greater in comparison with hybrid rye flours and smaller in comparison with flours made from open-pollinated rye (Table 2). Wholemeal flour from primitive rye was characterized by a higher proportion of fine particles (d(0.1) = 18.4 µm) and a higher relative span of volume-based size distribution (SPAN) than flours from the reference rye cultivars. Extracted flour from primitive rye was characterized by a smaller proportion of fine particles (d(0.1) = 14.5 µm) and lower SPAN values than hybrid rye flour, whereas the reverse was noted in comparison with the open-pollinated cultivar. The granulometric composition of flour significantly influences rheological and end-product properties (Bucsella et al. 2016). A higher proportion of fine flour particles intensifies dough fermentation because enzymes have easier access to starch and pentosans.

**Table 2:** Particle size characteristics of extracted and wholemeal flours made from primitive rye and common rye grain

Flour type	Genotype/cultivar	*d*_*avg*_ (µm)	*d(0.1)* (µm)	*d(0.5)* (µm)	*d(0.9)* (µm)	SPAN (−)
Extracted flour	Primitive rye	73.8^b^ ± 0.7 **	14.5^b^ ± 0.3	54.6^b^ ± 1.1	220.6^a^ ± 2.9	3.78^b^ ± 0.04
	Dańkowskie Złote (OP*)	80.0^c^ ± 0.3	17.6^c^ ± 0.2	67.5^c^ ± 0.6	235.6^b^ ± 4.2	3.23^a^ ± 0.06
	KWS Bono (H)	71.0^a^ ± 0.5	13.2^a^ ± 0.4	50.8^a^ ± 0.8	220.5^a^ ± 3.1	4.08^c^ ± 0.06
Wholemeal flour	Primitive rye	74.8^b^ ± 1.2	18.4^a^ ± 1.3	245.2^a^ ± 37.6	938.3^a^ ± 82.2	3.78^a^ ± 0.25
	Dańkowskie Złote (OP)	84.2^c^ ± 0.9	24.1^b^ ± 1.3	295.0^a^ ± 37.4	1123.2^b^ ± 101.0	3.74^a^ ± 0.22
	KWS Bono (H)	73.4^a^ ± 1.1	20.5^a^ ± 1.6	318.6^a^ ± 82.3	1080.0^b^ ± 97.2	3.43^a^ ± 0.51

*OP open-pollinated cultivar, *H* hybrid cultivar

**Values given as the average values ± SD. Values marked with the same letters in columns for the same type of flour are not significantly different at *p* ≤ 0.05

### Flour yield, flour quality characteristics and baking quality

The quality characteristics of extracted and wholemeal flours from primitive rye and common rye grain are presented in Table 3. The extraction rate of primitive rye flour (49.8%) was similar to that of hybrid rye flour (48.9%), and significantly lower than the extraction rate of flour from open-pollinated rye (53.5%). Rye flour has a lower extraction rate than wheat flour because the endosperm is difficult to separate from the seed coat, especially in grain with a high content of non-starch polysaccharides. The surface of sifting rye flour is 25–30% larger than required for wheat flour (Arendt and Zannini 2013).

**Table 3:** Quality characteristics of extracted and wholemeal flours from primitive rye and common rye grain

Quality parameter	Flour type					
	Extracted flour			Wholemeal flour		
	Primitive rye	Dańkowskie Złote (OP*)	KWS Bono (H)	Primitive rye	Dańkowskie Złote (OP)	KWS Bono (H)
Flour yield (%)	49.8^a^ ± 1.2**	53.5^b^ ± 1.3	48.8^a^ ± 1.3	–	–	–
Protein (% DM)	5.91^b^ ± 0.04	4.78^a^ ± 0.06	5.82^b^ ± 0.14	9.59^b^ ± 0.24	7.95^a^ ± 0.02	9.68^b^ ± 0.16
Ash (% DM)	0.59^c^ ± 0.02	0.53^b^ ± 0.01	0.46^a^ ± 0.01	1.92^c^ ± 0.01	1.61^b^ ± 0.02	1.41^a^ ± 0.02
Starch (% DM)	64.9^a^ ± 1.2	69.6^b^ ± 0.0	76.9^c^ ± 0.0	54.9^a^ ± 0.6	57.1^b^ ± 0.6	57.8^b^ ± 0.6
Color parameters						
L*	94.1^c^ ± 0.07	93.8^b^ ± 0.11	93.6^a^ ± 0.06	86.9^a^ ± 0.6	85.8^a^ ± 0.9	86.3^a^ ± 1.3
a*	− 0.10^c^ ± 0.2	− 0.53^a^ ± 0.02	− 0.23^b^ ± 0.02	1.06^b^ ± 0.11	0.72^a^ ± 0.16	0.55^a^ ± 0.12
b*	5.18^a^ ± 0.14	5.95^b^ ± 0.08	5.0^a^ ± 0.16	6.25^b^ ± 0.30	6.62^b^ ± 0.51	5.66^a^ ± 0.30
Falling number (s)	137^a^ ± 4	179^b^ ± 6	277^c^ ± 12	129^a^ ± 1	160^b^ ± 5	268^c^ ± 7
Initial gelatinization temp. (°C)	48.8^b^ ± 0.0	47.8^a^ ± 0.2	51.0^c^ ± 0.0	51.5^a^ ± 0.2	51.0^a^ ± 0.0	53.7^b^ ± 0.4
Final gelatinization temp (°C)	64.1^a^ ± 0.4	64.9^a^ ± 0.4	78.8^b^ ± 0.8	63.8^a^ ± 0.1	65.4^b^ ± 0.2	75.4^c^ ± 0.4
Amylograph peak viscosity (AU)	610^a^ ± 10	670^b^ ± 15	945^c^ ± 5	288^a^ ± 3	293^a^ ± 3	465^b^ ± 5
Water absorption (%)	53.2^b^ ± 0.4	51.3^a^ ± 0.7	59.6^c^ ± 0.2	55.6^b^ ± 0.5	53.1^a^ ± 0.5	65.2^c^ ± 0.6

*OP open-pollinated cultivar, *H* hybrid cultivar

**Values given as the average values ± SD. Values marked with the same letters in rows for the same type of flour are not significantly different at *p* ≤ 0.05

The protein content of wholemeal flour (9.59%) and extracted flour (5.91%) from primitive rye grain was significantly higher in comparison with the corresponding flours from open-pollinated rye and was similar to hybrid rye flours. The protein content of primitive rye flours was typical of rye flour (Konopka et al. 2017b; Järvan et al. 2018). The total protein content of rye is generally lower in comparison with wheat (6.5–14.5% on average) (Arendt and Zannini 2013). The ash content of wholemeal (1.92%) and extracted (0.59%) flours from primitive rye was significantly higher in comparison with flours made from open-pollinated and hybrid cultivars (wholemeal flour—higher by 16% and 27%; extracted flour—higher by 10% and 22%, respectively). The starch content of wholemeal flour and extracted flour from primitive rye (54.9% and 64.9%, respectively) was considerably lower relative to the corresponding flours from the reference rye cultivars. Similar observations were made by Konopka et al. (2017b).

The color analysis revealed that unlike wholemeal flours, extracted flours differed significantly in lightness (Table 3). The value of L* was highest in extracted flour from primitive rye grain. Both wholemeal and extracted flours from primitive rye were also characterized by the highest contribution of redness, which could be attributed to their high ash content. The value of the correlation coefficient between ash content and a* was determined at R = 0.98. Flour whiteness is determined by the values of L* and a*, and it is an important parameter which influences consumer acceptance. The most desirable flours are characterized by low values of a* and high values of L* (Drakos et al. 2017). Flour lightness is correlated with its ash content (Protonotariou et al. 2014). High ash content increases contamination with seed coat residues and decreases the value of L*. Particle size can also influence the color of flour. Flours with a higher proportion of fine particles are characterized by higher L* values (Gómez et al. 2009). The falling number denotes the presence of α-amylases in flour, and it was determined at 129 s in wholemeal flour and 137 s in extracted flour from primitive rye (Table 3). The falling number of both extracted and wholemeal flours from primitive rye grain was lower in comparison with the corresponding flours from the remaining rye cultivars. The extraction process clearly influenced the falling number, and wholemeal flours were characterized by lower falling numbers. In sourdough bread, a low falling number is more desirable due to high amylolytic activity which rapidly initiates the fermentation process (Zieliński et al. 2008). In the current study, primitive rye flour was most suitable for the production of sourdough bread. In wholemeal and extracted flours from primitive rye, starch gelatinization began at a temperature of 51.5 °C and 48.8 °C and was completed at 63.8 °C and 64.1 °C, respectively. In wholemeal and extracted flours from primitive rye, the temperature marking the beginning and end of starch gelatinization was 2.2 °C and 11.6 °C lower and 2.2 °C and 14.7 °C lower, respectively, than in the corresponding flours from rye cv. KWS Bono. Starch gel viscosity in primitive rye flour (288 in wholemeal flour and 610 in extracted flour) was lower than in flours from open-pollinated and hybrid ryes. Wholemeal and extracted flours differed significantly in starch viscosity. Wholemeal flours were less viscous, and similar results were noted in an earlier study (Konopka et al. 2017b). The optimal parameters of rye flour for breadmaking are determined in the following range: falling number: 125–200 s, viscosity: 400–600 AU, final starch gelatinization temperature: 63–68 °C (Beck et al. 2011; Konopka et al. 2017b). In view of the above, the analyzed flour from primitive rye grain was characterized by high baking quality, but its maximum amylograph viscosity was lower in comparison with the remaining rye flours, which can decrease bread volume (Stępniewska et al. 2018). The water absorption capacity of wholemeal and extracted flours from primitive rye grain was higher relative to the corresponding flours from open-pollinated rye, but lower in comparison with hybrid rye flours. The hydration capacity of flour is affected by its protein and polysaccharide content, degree of milling and starch damage (Drakos et al. 2017). Finely ground flour has a higher water absorption capacity.

### Content of free phenolic compounds and antioxidant potential

Rye flour is a rich source of phenolic compounds (Zieliński et al. 2008; Pejcz et al. 2015; Konopka et al. 2017b; Pihlava et al. 2018) which deliver health benefits due to their antioxidant potential (Pejcz et al. 2015). The content of phenolic compounds and the antioxidant activity of flour are altered during baking. The relevant changes are induced by numerous factors, including fiber content, recipe, fermentation, baking process and the formation of Maillard reaction products (Banu et al. 2010; Konopka et al. 2014; Pejcz et al. 2015). The content of free phenolics and the antioxidant potential of extracted and wholemeal flours from primitive rye and common rye are presented in Table 4. The content of free phenolics ranged from 8.31 to 12.6 mg/100 g DM in samples of extracted flours, and from 48.16 to 58.57 mg/100 g DM in samples of wholemeal flours. Primitive rye flours were most abundant in free phenolics whose content was approximately 4.7 times higher in wholemeal flour than in extracted flour. Similar results were reported by other authors (Konopka et al. 2014). However, the above findings do not correspond to the antioxidant activity of primitive rye flour. The DPPH radical scavenging activity of extracted flour from primitive rye (52.7 µM TE/100 g) was approximately two-fold higher than in flour from open-pollinated rye, and comparable with hybrid rye flour. The DPPH radical scavenging activity of wholemeal flour from primitive rye (200 µM TE/100 g) was similar to that noted in the corresponding flour from open-pollinated rye (186.9 µM TE/100 g) and lower than in hybrid rye flour (235.7 µM TE/100 g). The analyzed parameter was approximately 3.8-fold higher (primitive rye), 8-fold higher (open-pollinated rye) and 4.2-fold higher (hybrid rye) in wholemeal flours than in extracted flours. Zieliński et al. (2008) observed a reverse trend in rye flours in the DPPH test. A positive correlation was observed between the content of free phenolic compounds and the antioxidant potential of the analyzed flours (R = 0.95).

**Table 4:** Content of free phenolic compounds and the antioxidant potential of extracted and wholemeal flours from primitive rye and common rye

Flour type	Genotype/cultivar	Free phenolics (mg/100 g of DM)	DPPH (µM TE/100 g of DM)
Extracted flour	Primitive rye	12.6^b^ ± 0.8**	52.7^b^ ± 1.5
	Dańkowskie Złote (OP*)	9.3^a^ ± 0.4	23.4^a^ ± 2.4
	KWS Bono (H)	8.3^a^ ± 0.7	55.6^b^ ± 6.8
Wholemeal flour	Primitive rye	58.6^c^ ± 0.6	200.0^a^ ± 6.7
	Dańkowskie Złote (OP)	50.9^b^ ± 1.1	186.9^a^ ± 1.0
	KWS Bono (H)	48.2^a^ ± 1.1	235.7^b^ ± 18.0

*OP open-pollinated cultivar, *H* hybrid cultivar

**Values given as the average values ± SD. Values marked with the same letters in columns for the same type of flour are not significantly different at *p* ≤ 0.05

## Conclusion

A thorough knowledge of the attributes of primitive rye grain is essential for obtaining food products of the highest quality. The results of this study indicate that primitive rye (*S. cereale* var*. Multicaule*) has shorter kernels, lower thousand-kernel weight and a higher contribution of redness in color compared with common rye grain. Primitive rye grain is suitable for milling. Its mechanical properties, milling energy, flour yield and the granulometric composition of flours are typical of common rye grain. Both extracted and wholemeal flours of primitive rye grain contain more ash and less starch than common rye flours. The results of the amylograph test revealed that primitive rye flours were characterized by high baking quality. Primitive rye flours contain more free phenolic compounds than common rye flours, and their antioxidant potential is comparable with that of the reference flours. All of the analyzed wholemeal flours were characterized by higher DPPH radical scavenging activity than extracted flours.

The processing suitability of the grain and flours of primitive rye was similar to that of the grain and flours of organically-grown common rye (open-pollinated and hybrid cultivars). However, further research is needed to analyze variations in the properties of primitive rye grain and flour resulting from changes in environmental and climatic conditions.

## References

[CR1] American Association of Cereal Chemists (2002). Approved methods of the AACC.

[CR2] Arendt EK, Zannini E, Arendt EK, Zannini E (2013). Rye. Woodhead publishing series in food science, technology and nutrition, cereal grains for the food and beverage industries.

[CR3] Banu I, Vasilean I, Aprodu I (2010). Effect of lactic fermentation on antioxidant capacity of rye sourdough and bread. Food Sci Technol Res.

[CR4] Beck M, Jekle M, Selmair PL, Koehler P, Becker T (2011). Rheological properties and baking performance of rye dough as affected by transglutaminase. J Cereal Sci.

[CR5] Bucsella B, Molnár D, Harasztos AH, Tömösközi S (2016). Comparison of the rheological and end-product properties of an industrial aleurone-rich wheat flour, whole grain wheat and rye flour. J Cereal Sci.

[CR6] Dias VV, Schultz G G, Schuster MS, Talamini E, Révillion JP (2015). The organic food market: a quantitative and qualitative overview of international publications. Ambient Soc.

[CR7] Drakos A, Malindretou K, Mandala I, Evageliou V (2017). Protein isolation from jet milled rye flours differingin particle size. Food Bioprod Process.

[CR8] Dziki D, Cacak-Pietrzak G, Miś A, Jończyk K, Gawlik-Dziki U (2014). Influence of wheat kernel physical properties on the pulverizing process. J Food Sci Technol.

[CR9] Gómez M, Pardo J, Oliete B, Caballero P (2009). Effect of the milling processes on quality characteristic of rye flour. J Sci Food Agric.

[CR10] Hameed Hassoon W, Dziki D (2017) The study of multistage grinding of rye. In: Lorencowicz E, Uziak J, Huyghebaert B (eds) Farm machinery and processes management in sustainable agriculture, 9th international scientific symposium ULS Lublin, Poland, pp 125–129. 10.24326/fmpmsa.2017.23

[CR11] ICC (1990). ICC-standard no: 104/1. Determination of ash in cereals and cereal products.

[CR12] ICC (1992). Standard Method No. 115/1. Method for using the Brabender Farinograph.

[CR13] Järvan M, Lukme L, Tupits I, Akk A (2018). The productivity, quality and bread-making properties of organically and conventionally grown winter rye. Zemdirbyste.

[CR14] Jonsson K, Andersson R, Bach Knudsen KE, Hallmans G, Hanhineva K, Katina K, Kolehmainen M, Kyrø C, Langton M, Nordlund E, Lærke HN, Olsen A, Poutanen K, Tjønneland A, Landberg R (2018). Rye and health—where do we stand and where do we go?. Trends Food Sci Technol.

[CR15] Jouki M, Emam-Djomeh Z, Khazaei N (2012). Physical properties of whole rye seed (*Secale cereale*). IJFE.

[CR16] Koistinen VM, Mattila O, Katina K, Poutanen K, Aura AM, Hanhineva K (2018). Metabolic profiling of sourdough fermented wheat and rye bread. Sci Rep.

[CR17] Konopka I, Tańska M, Faron A, Stępień A, Wojtkowiak K (2012). Comparison of the phenolic compounds, carotenoids and tocochromanols content in wheat grain from organic and mineral fertilization. Molecules.

[CR18] Konopka I, Tańnska M, Faron A, Czaplicki S (2014). Release of free ferulic acid and changes in antioxidant properties during the wheat and rye bread making process. Food Sci Biotechnol.

[CR19] Konopka I, Grabiński J, Skrajda M, Dąbrowski G, Tańska M, Podolska G (2017). Variation of wheat grain lipid fraction and its antioxidative status under the impact of delayed sowing. J Cereal Sci.

[CR20] Konopka I, Tańska M, Skrajda M (2017). Evaluation of baking value and content of selected pro-healthy compounds in grain of primitive rye “krzyca”. Przegl Zboż-Młyn.

[CR21] Michalova A (2000) Review of minor cereals and pseudo-cereals in Europe. In: Maggioni L, Spellman O (compilers) Report of a network coordinating group on cereals. Ad hoc meeting. Radzików. Poland, 7–8 July 2000, p 41. http://www.ecpgr.cgiar.org/fileadmin/bioversity/publications/pdfs/19__Report_of_a_network_coordinating_group_on_cereals__ad_hoc_meeting__7-8_July_2000__Radzikow__Poland.pdf

[CR22] Pejcz E, Gil Z, Wojciechowicz-Budzisz A, Półtorak M, Romanowska A (2015). Effect of technological process on the nutritional quality of naked barley enriched rye bread. J Cereal Sci.

[CR23] Pihlava JM, Hellström J, Kurtelius T, Mattila P (2018). Flavonoids, anthocyanins, phenolamides, benzoxazinoids, lignans and alkylresorcinols in rye (*Secale cereale*) and some rye products. J Cereal Sci.

[CR24] PN-EN ISO 10520:2002 Skrobia naturalna—Oznaczanie zawartości skrobi—Metoda polarymetryczna Ewersa (English: Natural starch—Determination of starch content—Polarimetric method Ewersa)

[CR25] PN-EN ISO 3093: 2010 Pszenica, żyto i mąki z nich uzyskane, pszenica durum i semolina - Oznaczanie liczby opadania metodą Hagberga-Pertena (English: Wheat, rye and flours obtained from them, durum wheat and semolina—determination of falling number using the Hagberg-Perten method)

[CR26] PN-EN ISO 712: 2012 Ziarno zbóż i przetwory zbożowe—Oznaczanie wilgotności—Metoda odwoławcza (English: Cereal grains and cereal preparations—determination of moisture–appeal method)

[CR27] PN-EN ISO 7973: 2016-01 Ziarno zbóż i przetwory zbożowe—Oznaczanie lepkości mąki—Metoda z zastosowaniem amylografu (English: Cereal grains and cereal preparations—determination of stickiness of flour—method using an amylograph)

[CR28] Podyma W, Kuszewska K, Tyburski J, Tyburski J, Kostrzewska M (2013). Characteristics and possibility of utilization of currently not valued cereals. Biological diversity of agro-ecosystems and possibility of its protection on organic farms (In Polish).

[CR29] Ponce-García N, Ramirez-Wong B, Escalante-Aburto A, Torres-Chavez PI, Figueroa J, El-Amin MF (2016). Mechanical properties in wheat (*Triticum aestivum*) kernels evaluated by compression tests: a review. Viscoelastic and viscoplastic materials.

[CR30] Protonotariou S, Drakos A, Evageliou V, Ritzoulis C, Mandala I (2014). Sieving fractionation and jet mill micronization affect the functional properties of wheat flour. J Food Eng.

[CR31] Rydzak L, Andrejko D, Sagan A, Nakonieczny P (2012). The influence of vacuum impregnation and infrared radiation heat treatment of rye grain on energy consumption of the milling process. Inżynieria Rolnicza.

[CR32] Stępniewska S, Słowik E, Cacak-Pietrzak G, Romankiewicz D, Szafrańska A, Dziki D (2018). Prediction of rye flour baking quality based on parameters of swelling curve. Eur Food Res Technol.

[CR33] Vejražka K, Faměra O, Kocourková Z (2011) Selected grain physical properties of different rye varieties (*Secale cereale*) and milling quality. In: Proceedings of the 6th international congress flour-bread ‘11. 8th Croatian Congress of Cereal Technologists, Opatija, Croatia, 12–14 Oct 2011, pp 461–466 https://www.cabdirect.org/cabdirect/FullTextPDF/2012/20123355674.pdf

[CR34] Warechowska M, Markowska A, Warechowski J, Miś A, Nawrocka A (2016). Effect of tempering moisture of wheat on grinding energy, middlings and flour size distribution, and gluten and dough mixing properties. J Cereal Sci.

[CR35] Zieliński H, Michalska A, Ceglińska A, Lamparski G (2008). Antioxidant properties and sensory quality of traditional rye bread as affected by the incorporation of flour with different extraction rates in the formulation. Eur Food Res Technol.

[CR36] Zykin PA, Andreeva EA, Lykholay AN, Tsvetkova NV, Voylokov AV (2018). Anthocyanin composition and content in rye plants with different grain color. Molecules.

